# Phytopharmacological Possibilities of Bird Cherry *Prunus padus* L. and *Prunus serotina* L. Species and Their Bioactive Phytochemicals

**DOI:** 10.3390/nu12071966

**Published:** 2020-07-02

**Authors:** Aleksandra Telichowska, Joanna Kobus-Cisowska, Piotr Szulc

**Affiliations:** 1 Faculty of Food Science and Nutrition, Poznan University of Life Sciences, Wojska Polskiego 28, 60-637 Poznan, Poland; joanna.kobus-cisowska@up.poznan.pl; 2 Foundation for the Education of Innovation and Implementation of Modern Technologies, Widok 11, 62-069 Dabrowka, Poland; 3 Department of Agronomy, Poznan University of Life Sciences, Wojska Polskiego 28, 60-637 Poznan, Poland; piotr.szulc@up.poznan.pl

**Keywords:** *Prunus padus*, *Prunus serotina*, bird cherry, phytochemical compounds, cyanogenic glycosides, antioxidant, antibacterial, anti-inflammatory

## Abstract

Wild cherry is a plant observed in the form of trees or shrubs. This species comprises about twenty kinds of plants and the most popular are two, *Prunus padus* L. and *Prunus serotina* L., whose properties and content of phytochemical compounds are subject to studies. Wild cherry contains many active compounds, including tocopherols, vitamins, polyphenols and terpenes, which can have beneficial effects on health. On the other hand, wild cherry contains cyanogenic glycosides. Nevertheless, current research results indicate pro-health properties associated with both *P. serotina* and *P. padus*. The aim of this study was to collect and present the current state of knowledge about wild cherry and to review available in vitro and in vivo studies concerning its antioxidant, anti-inflammatory, antibacterial and antidiabetic activity. Moreover, the current work presents and characterizes phytochemical content in the leaves, bark and fruits of *P. padus* and *P. serotina* and compiles data that indicate their health-promoting and functional properties and possibilities of using them to improve health. We find that the anatomical parts of *P. padus* and *P. serotina* can be a valuable raw material used in the food, pharmaceutical and cosmetic industries as a source of bioactive compounds with multi-directional action.

## 1. Introduction

Currently, there is a growing interest in the possibilities of an application of plant raw materials in medicine as well as in functional food designing. The biological activity of raw materials depends on the content of individual phytochemicals, which are found in different anatomical parts of plants: fruits, shoots, buds, leaves, roots and bark [1,2].

One of these plants is wild cherry. The species comprises about twenty kinds of plants, however, two of them are the most popular: European bird cherry (*P. padus* L. syn. *Prunus avium* L.) and American black cherry (*P. serotina* L.) [3] The ecological expansion of the wild cherry tree results from its high efficiency of vegetative multiplication and generative propagation due to features such as its high capacity and speed of seed germination, high seed production, long dispersion, rapid growth and development, low requirements in relation to habitat conditions and high tolerance to climatic conditions [4].

The wild cherry is mistakenly considered inedible. The fruit seeds contain cyanogenic glycosides, the excessive consumption of which, without thermal treatment, may have adverse health effects [5]. The consumption of wild cherry already took place in prehistoric times. This is evidenced by the remains of Neolithic and bronze vessels, the decorations of which depict the wild cherry, which were discovered in palafittes (houses erected on stilts) in South-East Asia. The Greek historian Herodotus of Halicarnassus also informs about its use. At that time, the fruit was consumed with the addition of salt, and regardless of this, alcohol was also produced from it [6].

Currently, the European bird cherry (*P. padus*) and the American black cherry (*P. serotina*) are not widely used in pharmaceutical or food industries. The aim of this study is to characterize the phytochemical compounds present in wild cherries, their biological properties and the possibility of their use in the pharmaceutical, food and cosmetics industries on the basis of available literature data. This review encompassed research conducted at the in vitro (outside of the normal biological context, commonly called test-tube experiments) and in vivo (experiments developed using the whole organism) levels. Additionally, the authors present the data from all available papers, in a comprehensive way, providing complete information about the main analytical parameters. Although many researchers have reported the effectiveness of wild cherry in in vitro and in vivo studies, more work needs to be done to clinically prove their effectiveness.

## 2. Data Collections

All data presented in this review were summarized from the references, including scientific journals, book chapters or dissertations. These references were systematically searched against electronic databases: PubMed, CNKI (http://new.oversea.cnki.net/index/), Web of Science, Scopus and Google Scholar and SciELO with a keyword “*Prunus padus*” and “*Prunus serotina*”. To search for maximum relative references, the keyword was set as “bird cherry” without any other restrictions. Subsequently, references closely related to chemical compositions, traditional uses and pharmacological properties, including in vitro and in vivo investigations, were screened for further data extraction. In addition, to survey the taxon, phenotypes and geographical distributions of *Prunus* species, several online taxonomic databases, including http://theplantlist.org/, http://www.worldfloraonline.org/, https://www.gbif.org/ and http://www.iplant.cn/foc/, were also explored.

## 3. Habitats and Classification

Wild cherry (*Prunus* L.) is a type of tree or large shrub belonging to the subfamily *Amygdaloideae* (*Prunoideae*) within the *Rosaceae* family (Table 1). This subfamily also includes three other genera which are distinguished by their drupe-shaped fruit [7,8].

The classification of the species *Prunus* L. has always been controversial and has been presented differently by many authors. In 1700, the French botanist Joseph Pitton de Tournefort was the first to classify the distinguished species into six genera, distinguishing them on the basis of differences in fruit structure: *Amygdalus*, *Armeniaca*, *Cerasus*, *Laurocerasus*, *Persica* and *Prunus*. This classification was adopted in 1754 by the Swedish naturalist Carl Linnaeus, who distinguished four genera: *Amygdalus*, *Cerasus*, *Prunus* and *Padus*.

The idea of merging all the species was first published by George Bentham and Joseph Dalton Hooker in 1865 [7,9]. Rehder’s classification from 1940 of five subgenres within *Prunus* gained the widest acceptance: *Amygdalus*, *Cerasus*, *Laurocerasus*, *Padus* and *Prunus* [7,10].

Recent DNA tests have shown that *Prunus* is monophilic and derived from some Eurasian ancestors [8]. Plants of the *Prunus* species mainly occur in the northern hemisphere and the temperate zone, most often in Europe, Asia Minor and Western Siberia [11]. Wild cherry grows well in partially darkened sites and mineral-rich soils, and its location does not affect the characteristics of trees such as leaves, fruits or bark [6].

## 4. Botanical Characteristics of *P. padus* and *P. serotina*

The height of trees of the bird cherry tree (*P. padus*) can reach up to 15 m [12], while shrubs range from 0.5 to 4 m (Table 2). American black cherry trees (*P. serotina*) can reach a height of up to 25 m [13]. Its structure consists of a round or egg-shaped crown with falling branches and dark brown shoots, which form a shrubby form [14,15]. The trees reach their target dimensions at the age of 100 years [16].

The distinction between the bird cherry *P. padus* and the American black cherry *P. serotina* is possible due to the shape of the leaves and flowers. The American black cherry is characterized by elongated, lancet, pointed leaves, which are sharply serrated at the edges and are shiny and dark green on the surface. They are light green on the underside [17,18]. They are characterized by a poorly visible vein network. The leaves of the bird cherry are smaller and its edges are finely serrated. The petiole is usually red and the leaves themselves are more matt and leathery. The vein network is dense, connected at the edge of the leaf [15].

The bird cherry begins to flower at the end of April and the beginning of May, while the American black cherry only flowers around May and June. The flowers are creamy white and form cylindrical clusters [19,20]. The flowers of American black cherry do not have an aroma and are smaller than those of the bird cherry [21]. Small, spherical black drupes are fruits that appear around August and September [22,23].

## 5. Nutrition Value and Mineral Content of *P. padus* and *P. serotina*

Natural products, mainly of plant origin, are gaining interest all over the world. Some of them are already used as a substitute or complementary to unconventional forms of drugs due to the toxicity and side effects of synthetic drugs [24,25,26,27,28,29].

The analysis of the chemical composition of leaves, bark, flowers and fruits of wild cherry trees has been the subject of many studies concerning selected traits or species of wild cherry (Table 3).

Table 3 shows the nutritional value of the fruits of the *P. padus* and *P. serotina* cherries. Fruits of *P. serotina* contain a relatively high content of protein and a high content of carbohydrates [31]. The protein content in fruits of *P. serotina* is higher than reported in the literature concerning plums (0.9% ± 0.03%), apricots (*Prunus armeniaca* L.) (1.4% ± 0.33%), peaches (*Prunus persica* L. Batsch) (0.9% ± 0.01%) or grapes (0.72% ± 0.03%) [33].

A higher carbohydrate content was noted in the fruit of *P. padus* [30,32]. It was also found to contain reducing sugars such as glucose, fructose and sorbitol [30]. Sorbitol is also found in other fruits besides wild cherries and is present in relatively high amounts in apples, peaches, apricots and in small amounts in gooseberries, currants, bananas, pineapples, but also in rowanberries [34,35,36,37]. The mineral components found in the seeds of *P. serotina* are mainly: iron, magnesium, potassium, zinc, phosphorus and sodium. The content of magnesium in raw and roasted seeds of *P. serotina* was not affected by heat treatment, but the content of Ca, Fe, P, K, Zn and Na decreased after the seeds were roasted. Raw wild cherry seeds also contain a relatively high protein content (37% ± 0.16%). It was shown that the content of amino acids in roasted seeds increased [32]. The content of fiber in the seeds depended on the heat treatment and its highest content was found in the roasted seeds of *P. serotina* [32].

## 6. Phytocompounds Content in *P. padus* and *P. serotina*

### 6.1. Chlorophyll and Anthocyanins

Chlorophyll absorbs energy from light, which is used to convert carbon dioxide into carbohydrates [38]. Chlorophyll occurs in several different forms [39,40]. The main types are chlorophylls A and B. In the fruit of *P. padus* there is both chlorophyll A and chlorophyll B, but there are twice as many chlorophyll A (Table 4).

Wild cherries also contain carotenoids—most of them are beta-carotene [30]. Recently, there has also been a significant increase in interest in possible pro-health effects associated with the consumption of anthocyanins. It is suggested that fruits rich in anthocyanins or their extracts show a wide range of protective effects [43,44]. The consumption of anthocyanins prevents the risk of cardiovascular diseases, oxidative stress and diabetes [43,44,45,46]. The antioxidant activity of cyanidin is 4.4 times higher than that of ascorbic acid. Among edible plants, berries of red, blue or purple color, such as wild cherries, are one of the most important sources of anthocyanins in the diet [5,47,48,49,50]. Anthocyanins are mainly present in the outer layers of subcutaneous tissue.

In *P. padus* fruits, the total content of anthocyanins was 2071.16 ± 91.02 mg/kg FW [30]. The main compounds in this group were mainly: cyanidin-3-glucoside, cyanidin-3-rutinoside as well as cyanidin-3-galactoside and cyanidin rhamnosyl hexoside [30].

### 6.2. Vitamin: Tocopherols and Vitamin C

Tocopherols are commonly found in cereals, vegetable oils and eggs [51,52]. These include alpha-tocopherol, beta-tocopherol, gamma-tocopherol and delta-tocopherol. However, alpha-tocopherol with antioxidant properties has the greatest nutritional importance [53]. Alpha, beta and gamma-tocopherol have been found in *P. padus* fruits, where alpha-tocopherol is dominant and constitutes as much as 63% of all tocopherols (Table 4) [30]. In raw seeds, alpha-tocopherol is present at a level of 3.916 mg / 100 g, while it is absent in roasted seeds [32,54].

The fruits of *P. padus* also contain vitamin C, in the form of ascorbic acid at a level of 25.20 ± 3.48 mg/100 FW and in the form of dehydroascorbic acid at a level of 50.87 ± 16.23 mg/100 FW [55,56]. Ascorbic acid is a saccharide derivative. It is produced from D-glucose in organisms of animals capable of synthesizing it [57]. In plants it is produced from D-glucose or D-galactose. Vitamin C is biologically active [58]. It takes part in many reactions and transformations, stimulating various biochemical processes in the body. Because of good solubility of vitamin C and active transport, about 80% is is absorbed by the organism [59,60]. The structure of ascorbic acid, which contains two adjacent groups, hydroxyl and carbonyl, makes this molecule an excellent hydrogen or electron donor, which determines the antioxidant activity. Ascorbic acid can be a donor of two electrons and therefore takes part as a cofactor in many enzymatic reactions taking place in the body.

### 6.3. Terpens

Non-glycosylated pentacyclic triterpenoids such as ursolic acid (3b-hydroxyurs-12-en-28-ic acid), corosolic acid (2a, 3b-dihydroxyurs-12-en-28-ic acid) and oleanolic acid (3b-hydroxyolean-12-en-28-ic acid) are lipophilic constituents, which have recently aroused great interest in the context of phytotherapy because of their comprehensive biological properties, including anti-inflammatory, anti-ulcer, antioxidant, hepatoprotective, anticancer, antiatherosclerotic and antidiabetic ones [61,62,63,64,65,66].

These acids are widely distributed in various medicinal plants and plants of the *Prunus* species (*Rosaceae*). It was found that leaves and whole inflorescences of *P. padus* were the richest source of triterpenic acids (Table 4). It was shown that during the entire vegetation season, the highest levels of triterpenes were accumulated in the leaves between September and October, which are therefore recommended as the optimal time to harvest high quality plant material [67]. Terpenes such as limonene, phellandrene, sabinene, γ-terpinene, ursolic acid, uvaol, cis-linalool oxide, trans s-linalool oxide, (Z)-8-hydroxylinalool, E)-β-Farnesene or (E,E)-α-Farnesene were also found in wild cherry fruits [42].

### 6.4. Organic Acids

The plant raw materials also contain malic acid, citric acid and tartaric acid [68,69,70]. In the fruits they are mostly observed in free form, unlike in case of vegetables, where organic acids are bound. Malic and citric acid were found to be the main organic acids in wild cherry fruits (*P. padus*) (Table 5). In the fruit of *P. padus*, the highest content among the determined organic acids is noted for quinic acid and citric acid [55] and the lowest for shikimic acid [30]. There are also such organic acids as oxalic acid, malic acid or fumaric acid [30].

### 6.5. Polyphenolic Compounds in Wild Cherries (P. padus and P. serotina)

Phytochemical analysis showed that the main components of fruits (*P. padus*) are organic acids (48.62 ± 2.31%), polyphenols (35.34 ± 1.80%), monoterpenes (9.36 ± 0.64%) and vitamin C (6.68 ± 0.22%) (Table 5). Polyphenols present in *Prunus* are mainly: cinnamic acid, flavonols, benzoic acids, catechins and tannins [55]. The most important flavonols determined in the fruit were quercitrin (16.37 ± 3.51 mg/100 g FW) and quercetin (11.86 ± 2.36 mg/100 g FW).

Research on the influence of dietary polyphenols on human health has developed significantly in the last 10 years. They have been shown to be effective in the prevention of degenerative diseases, especially cardiovascular diseases and cancer [72,73]. The antioxidant properties of polyphenols have been extensively studied, but the health effects of polyphenols depend on the amount consumed and their bioavailability [74].

In fruits of *P. padus*, the presence of mainly caffeic acid, chlorogenic acid, coumaric acid and ferulic acid was found, of which coumaric acid was dominant [55,75] as well as 5-caffeolquinic acid 2 [30]. The content of ellagic acid, gallic acid and vanillic acid was also determined [55].

Flavonols, in turn, are among the most numerous plant polyphenols [76]. Their group is extremely diverse. The main share among flavonols in berries constitute quercetin derivatives [30]. It was demonstrated that epicatechin and catechin dominate among flavonols in *P. padus*. The content of flavanols in the fruits of *P. padus* also consist of quercetin and its derivatives, hyperoside, kaempferol and isorhamnetin. Kaempferol glycosides accounted for only 2%, and isorhamnetin glycosides were detected in amounts below 1 mg/kg FW [55].

According to Hertog et al., 1992 [77], a proper diet should provide plant polyphenols. The fruits of *P. padus* were shown to have a high flavonol content of 382 mg/kg FW compared to other vegetables and fruits [77]. *P. padus* can therefore be considered as a source of flavonols and used as a food ingredient or raw material for the production of supplements. Other studies demonstrated that flavones, like flavonols, are of significant biological importance [78]. Among flavones, apigenin rhamnoside in amounts of 24.99 ± 1.61 mg/kg FW was identified in the fruits of *P. padus* [30].

Another ingredient found in wild cherry are tannins. The content of condensed tannins in the fruit is mainly influenced by the environmental conditions of plant growth and the degree of fruit ripeness. Fruits of *P. padus* are characterized by a high tannin content of 5356 mg/kg FW [30]. It was demonstrated that tannins in the diet support the treatment of diabetes and have an anti-inflammatory effect [79].

## 7. Cyanogenic Glycosides: Amygdalin and Prunazine

The *P. serotina* plant is considered an invasive species. It contains cyanogenic glycosides such as prunazine and amygdalin. Their presence in the plant is considered to protect it from herbivores and pathogens [80]. Cyanogenic glycosides are produced by plants and are potentially toxic to herbivores due to the hydrolytic release of hydrocyanic acid [81].

The content of prunazine and amygdalin was determined in the fruits of wild cherry and its amount was compared to other raw materials (Table 6). It was shown that the leaves of the American black cherry (*P. serotina*) contain both these glycosides in amounts of 59.49 ± 1.31 mg/g ± SD prunazine and 20.95 ± 0.25 mg/g ± SD amygdalin [82]. These concentrations are high in comparison with other raw materials where the amygdalin content is lower, e.g., in *P. amygdalus* Batsch fruit oil, was 6.37 mg/g [83], in *Eriobotrya japonica* Lindl. flowers it was 50.76 ± 0.92 µg/mL [84]. In other fruits and vegetables the amygdalin content is as follows: green plum 17.49 ± 0.26 mg/g, apricot 14.37 ± 0.28 mg/g, black plum 10.00 ± 0.14 mg/g, peach 6.81 ± 0.02 mg/g, red cherry 3.89 ± 0.31 mg/g, black cherry 2.68 ± 0.02 mg/g, nectarine 0.12 ± 0.01 mg/g, courgette 0.21 ± 0.13 mg/g, cucumber 0.07 ± 0.02 mg/g and marrow 0.06 ± 0.01 mg/g [85].

## 8. Pharmacological Activity, Health-Promoting Properties

### 8.1. Antioxidant Activity

There are many studies available in the literature that indicate the high antioxidant potential of *P. padus* and *P. serotina* plants (Table 7). This concerns fruit as well as leaves, bark and flowers [31,55,87,88]. The studies were carried out in this range, where methanol extracts from the bark and leaves of *P. padus* were made and significant concentration-dependent antioxidant activity was demonstrated [88].

This activity was confirmed by 1,1-diphenyl-2-picrylhydrazine (DPPH) radical scavenging methods as well as by a reducing force. A positive correlation between antioxidant activity and flavonoid content was shown. Higher levels of flavonoid content were found in *P. padus* leaves (210.8 ± 2.6 μg QUE/g) compared to bark extract [88]. High antioxidant activity of aqueous extracts from fruits, flesh and peel of *P. serotina* was also shown by other authors. Higher activity of extracts prepared from the peel was also positively correlated with the presence of polyphenols [31]. These results are consistent with previous reports, where it was shown that *Prunus* fruit peels have at least a 20% higher polyphenol content than flesh [92,93]. This relationship also applies to other fruits such as apples and grapes [94,95]. Analyzing the data presented, it can be concluded that the antioxidant activity measured by DPPH and FRAP tests for whole fruits of *P. serotina* was higher than that obtained for plums or grapes by at least 15% [96,97].

These results indicate that wild cherry fruits can be a raw material with similar antioxidant activity as those considered as good sources of antioxidants. The content of polyphenols in the fruit peel of *P. serotina* correlated with the antioxidant activity of the fruits (r = 0.875 for DPPH and r = 0.959 for FRAP) [31]. However, such dependencies are not unequivocal. The content of polyphenols and their distribution in fruit depends largely on the ripening process and the genetic and environmental conditions [98].

Twelve phenolic compounds were found in the fruit peel of *P. serotina* which contribute to their strong antioxidant properties. The HPLC-MS analysis showed that cyanidin-3-O-rutinoside, chlorogenic acid, hyperoside and quercetin pentoside are present in the greatest amount in the *P. serotina* peel. However, the main phenolic compounds in the fruit flesh are cyanidin-3-O-rutinoside, chlorogenic acid, procyanidin B, hyperoside and quercetin malonilglucoside [31].

In another study, the antioxidant activity and polyphenol content in acetone and aqueous extracts from fruits of *Prunes padus* L was examined. The total polyphenol content was 11053.3 ± 491.28 mg GAE/kg FW fruit weight and the antioxidant activity (FRAP) 31.54 ± 0.26 mM trolox/kg [30]. Another study on fresh fruits of *P. padus* confirmed antioxidant properties and high content of polyphenols [55]. Extracts from fresh fruit were characterized by their ability to chelate metals at the level of 17.78 ± 0.84 mmol Fe^2+^ / kg FW. In another study, methanol extracts of flowers and leaves of *P. padus* were compared for antioxidant activity using DPPH radical. The aim of the comparison was to assess the activity of leaves collected at the turn of May and June and autumn leaves collected at the turn of July and August. It was shown that the highest antioxidant activity was observed in flowers, whose antioxidant activity was 1.43–1.49 g g^−1^ DPPH, as well as in leaves harvested in autumn, giving a value of 1.68 g^−1^ DPPH [71].

### 8.2. Antimicrobial Activity

The abuse of antibiotic therapy can have adverse effects [99,100,101,102]. Side effects of antibiotics such as immunosuppression, allergic reactions and hypersensitivity are known. Therefore, natural sources of bactericidal and bacteriostatic compounds in plants are sought [103]. Many substances contained in rhizomes, fruits, leaves or bark have been shown to have bactericidal effects [104,105,106,107,108].

The literature contains reports on the antibacterial effect of wild cherry components (Table 7). The beneficial effect of methanol extracts from seeds of *P. padus* on seventeen strains of pathogenic bacteria was demonstrated [89]. Earlier studies by the same authors confirmed the bactericidal effect of other methanol extracts obtained from the seeds of plants harvested in Scotland [109]. Methanol extracts obtained from seeds of *P. padus* were active against *Staphylococcus aureus*. In addition, the extracts also showed action against methicillin-resistant strains of *Staphylococcus aureus, Staphylococcus hominis* and *Proteus mirabilis*. Dichloromethane extracts, on the other hand, showed weak growth inhibition of *Enterococcus faecalis* and *Staphylococcus hominis* strains. The highest antibacterial activity was found for methanol extract from seeds of *P. padus* against *Staphylococcus aureus* (1.0 × 10^−4^ mg/mL) [89].

In another study, antimicrobial activity was assessed using methanol extracts from the leaves and branches of *P. padus*. Both leaf and branch extracts showed antimicrobial activity against most of the gram-positive bacteria tested, but only branch extract showed any activity against Gram-negative bacteria. *P. padus* branch extract was most active against *Kocuria rhizophila* (MIC = 125 μg/mL) [88].

### 8.3. Antidiabetic Effect

Many herbal preparations are used in the prevention and support of diabetes treatment [110]. It has been shown that alpha-glucosidase inhibitors of natural origin such as aqarbose,1-deoxynojirimycin and genistein, are beneficial in type 2 diabetes delaying the increase of blood glucose levels [111]. The literature points to such raw materials as: mulberry leaves [112,113], gurmar [114,115], Ceylon cinnamon [116,117], galega [118], common beans [119].

The beneficial antidiabetic effect was also confirmed for methanol extracts from the leaves and branches of *P. padus*, which were tested for their alpha-glucosidase inhibitory activity (Table 7). It was found that the extract from the branches showed higher activity, which was indicated by a higher content of polyphenols compared to the extract from leaves [88].

### 8.4. Cardiovascular Activity

One of the cardiovascular diseases is hypertension [120,121,122]. Currently, there is a search for plant preparations which action that will activate the main signaling pathways vasodilating endothelium [90]. In this respect, there are reports of beneficial effects of plant polyphenols in the literature. Positive effects in the prevention and treatment of hypertension have been found in the case of such plants as: *Nauclea latifolia* L. [123], green tea [124], grapefruit seeds [125], garlic [126] and globe amaranth (*Gomphrena globosa* L.) [127].

Studies on the influence of wild cherry on hypertension have confirmed that their efficacy is due to the presence of hyperoside and chlorogenic acid as smooth muscle relaxants in blood vessels (Table 7) [90]. Preparations made from the leaves and fruits of *P. serotina* may be a part of the diet supporting the treatment.

Other studies demonstrate that water and dichloromethane extracts from the fruit of *P. serotina* contain polar and non-polar vasodilating metabolites [31]. It was also noted that *P. serotina* fruits have a high content of phenolic compounds such as chlorogenic acid, gallic acid, coffee acid, catechin, epicatechin and quercetin and kaempferol glycosides, which are directly related to the high antioxidant activity and the resulting vasodilating effect [31]. These results were confirmed in another experiment where the effectiveness of wild cherry compounds in the H_2_S hydrogen sulfide pathway, which is responsible for vasodilation, was confirmed [31]. Available data indicate that H_2_S dilates vessels mainly by increasing the activity of ATP-sensitive potassium channels (KATP) in muscle cells. Triterpenes present in wild cherry fruits increased this activity. The study also confirmed the participation of wild cherry triterpenes in the NO/cGMP pathway. Moreover, it was shown that ursolic acid is able to activate both pathways simultaneously, which causes a synergic vasodilatatory effect. Studies in silico confirmed the beneficial effects of these compounds [90].

### 8.5. Anti-Inflammatory and Anti-Nociceptive Properties

Inflammatory process is an important defense mechanism in the human body. It may be characterized by redness, swelling, pain, feeling of warmth and dysfunction of tissue and organs [128,129,130,131]. In response to inflammation, the body activates cells in the immune response, which increase the production of pro-inflammatory mediators including nitrogen and prostaglandin. Earlier studies demonstrated that excessive production of nitrogen and prostaglandin takes part in inflammatory diseases, including rheumatoid arthritis, asthma and cancer [132,133]. Their high levels are produced by inductive enzymes such as inductive nitric oxide synthase and cyclooxygenase-2 [134]. Thus, methods are being sought to inhibit the expression of these enzymes, which may prove to be an attractive therapeutic agent in the treatment of inflammatory diseases [135]. Studies demonstrated that *P. padus* contains anthocyanins, cyanogenic glycosides, flavonoids and chlorogenic acid, which are important in the treatment of inflammation (Table 7) [71]. It also has antioxidant and antibacterial properties [89]. Methanol extracts from the stem of *P. padus* were analyzed, determining the anti-inflammatory and analgesic effects. In this study, a strong influence of the inhibitor on nitric oxide production induced by IFN-gamma/LPS was demonstrated. The extract suppressed not only the enzyme activity but also the expression of nitric oxide synthase. It also inhibited cyclooxygenase-2 expression depending on the dose. IFN-gamma/LPS stimulation induced NF-kB translocation to the nucleus, but was weakened by the presence of stem extract from *P. padus* [91]. The same study in vivo showed that the stem extract from *P. padus* may reduce paw swelling in six-week-old mice. It also shows a strong analgesic effect compared to tramadol and indomethacin. The study confirmed the strong anti-inflammatory properties of the extract not only by suppressing inflammatory mediators in vitro, but also by reducing inflammatory swelling in vivo. A strong anti-nociceptive effect through the central and peripheral mechanism acting as a partial opioid agonist was also proven. Based on these results, it can be suggested that *P. padus* has a strong anti-inflammatory and analgesic potential.

## 9. Cosmetic Properties of *P. padus* and *P. serotina*

Recently, the search for natural sources of active compounds, mainly of plant origin, and their beneficial properties has become a very clear trend in the cosmetics industry. Cosmetic companies are still looking for new natural ingredients that could enrich existing cosmetic compositions. Bark, leaves and fruits of *P. padus* are known for their anti-inflammatory, antimicrobial and antioxidant properties [91]. They contain polyphenols that indicate the potential of *Prunus* as a cosmetic ingredient. It is known that polyphenols have a strong antioxidant effect. Antioxidants delay the aging process of cosmetic products both during their use and storage. In practice, small amounts of antioxidants (0.001–0.01% by weight) can already deactivate free radicals and thus stop the chain oxidation of hydrocarbon lipid chains [136]. Phenolic contents in *P. padus* and *P. serotina* were very high compared to some known antioxidant plants such as thistle, mate, slippery elm bark or pine needles [137]. Polyphenols can also complement sun protection products and can be applied to treat inflammation caused by UV radiation, as well as to protect and prevent skin damage caused by oxidative stress or DNA damage.

*P. padus* fruits also contain a relatively high content of vitamin C, but it cannot be directly utilized to stabilize the oil phase of cosmetic products, as it is not soluble in fats. However, it is possible to conduct esterification with palmitic acid, and the obtained ascorbyl palmitate (ACP) is perfectly suited for use as an antioxidant both in cosmetic products and in the food industry.

Both *P. serotina* and *P. padus* fruits contained gallic acid. Antioxidants based on gallic acid are allowed in cosmetic products in weight concentrations from 0.005 to 0.01% [138]. Gallic acid (3,4,5-trihydroxybenzoic acid) esters are commonly used oil phase antioxidants for cosmetic preparations. Esters with low molecular weight alcohols and fatty acid esters are used to stabilize lipids, oils and cosmetic emulsions. It is also worth mentioning that in addition to antioxidant activity, gallic acid esters with long-chain alcohols also exhibit significant antifungal activity. A disadvantage of this group of antioxidants is that they cause unaesthetic yellow coloration of cosmetic preparations, especially in the presence of heavy metal cations [138].

Citric acid, present in *P. padus* fruits, also shows antioxidant activity; it binds heavy metal cations to non-dissociating complexes, thereby reducing the catalytic activity of metals in lipid auto-oxidation. Chlorogenic acid, which was also found in *P. padus* fruits, is listed among effective antioxidants as well. This compound binds iron ions, causing a loss of their ability to generate hydroxyl radicals, leading in turn to reduced lipid and protein peroxidation. It also prevents DNA damage induced by sunlight [139].

Essential oils from fruits, stems, leaves and bark of *P. padus* contain large amounts of benzoic acid and benzaldehyde. Benzoic acid has not only anti-bacterial, but also anti-fungal properties. This compound, already at a weight concentration of 0.1%, is an effective antimicrobial agent in an acidic environment, while at a weight concentration of about 1%, it can be added as a mild disinfectant, e.g., in ointments or mouth waters. The use of plant materials such as essential oils in cosmetic preparations at relatively high concentrations provide many benefits for the skin. They exhibit antimicrobial effects [140] and can serve as effective cosmetic preservatives. At present, synthetic preservatives are applied to extend product shelf life. However, while common synthetic preservatives extend the life of products and help keep them free from microorganisms, many of them hold a negative opinion in the minds of consumers, and their use has become increasingly controversial in recent years. Some of them can even cause serious health issues, e.g., 4-hydroxybenzoic acid esters, better known as parabens, were the most widely used preservatives (included in approximately 80% of global cosmetics [141]), but have recently been reported to mimic estrogen, increase female breast cancer incidence and influence the development of malignant melanoma. Essential oils can also be used as scents to enrich cosmetic fragrance.

The bark contains tannins, cerebrosides and polyphenols [71]. Due to the content of the above-mentioned bioactive substances, it seems appropriate to state that *P. padus* may be a good candidate for the production of natural cosmetics with strong effects.

The bark extract of *P. padus* was evaluated in terms of the use as a natural ingredient in cosmetics manufacturing. Extract from the bark of *P. padus* was obtained by extraction with hot water. The use of such an extract was evaluated for safety (cell toxicity test) and an effectiveness test (antioxidants, anti-wrinkle and whitening effect). The total concentration of polyphenols was 714.7 ± 0.5 mg/g and flavonoids 72.1 ± 2.2 mg/g. In comparison with other natural antioxidants, the concentration of polyphenols in the *P. padus* bark extract was very high. The extract showed 71% free radical scavenging activity of DPPH (antioxidant), 36% elastase inhibition (anti-wrinkle action) and 38% tyrosinase inhibition (whitening action) at 350 μg/mL. The preparation in the form of lotion, containing 1% of *P. padus* bark extract, showed that the viscosity, pH, particle size and appearance of the lotion remained stable for 28 days [142]. The lotion particle size was stable (362–426 nm) during stability tests. This study showed that the *P. padus* bark extract has promising potential as a natural antioxidant cosmetic agent.

## 10. Summary

In recent years there has been a great interest in products of plant origin and their health-promoting properties. Plants of *P. padus* and *P. serotina* are common in many parts of the world and do not require special cultivation conditions. The fruit, bark, leaves and flowers of the wild cherry are a good source of minerals, vitamins and compounds with high antioxidant activity, especially polyphenols.

Current scientific results confirm the beneficial properties of both *P. serotina* and *P. padus* extracts. They are attributed with a beneficial effect in the prevention of cardiovascular diseases and hypertension. Their antioxidant, anti-inflammatory, anti-nociceptive, antidiabetic and antimicrobial effects were also confirmed in the studies. Due to their content of anthocyanins, which show strong antioxidant properties, they can also be used in the pharmaceutical industry as a component of creams, gels and other care and disinfection preparations.

In summary, preparations from *P. padus* and *P. serotina* can be a valuable raw material used in the food, pharmaceutical and cosmetic industries as a source of bioactive compounds with multi-directional action.

## Figures and Tables

**Table 1:** Systematics of the species belonging to genus *Prunus* L.

Domain	Eukaryota
Kingdom	Plantae
Clade	Vascular plants
Clade	Seed plants
Class	Angiosperms
Clade	Rosids
Order	Rosales
Family	Rosaceae
Genus	Prunus

**Table 2:** Comparison of botanical characteristics of *P. padus* and *P. serotina* [6].

	*P. padus*	*P. serotina*
Country of origin	Europe, Asia Minor	North America
Height of trees	up to 15 m	up to 25 m
Leaves	Smaller, finely serrated	Elongated, sharpened, more serrated
Veins network (leaves)	Dense	Poorly visible
Fruits	Bitter	Sweeter
Flowering	April/May	May/June
Inflorescence	Suspended or rarely elevated	Elevated or ascending
Fruits	Petals broadly inversely egg-shaped, 10–15 mm long, almost twice as long as stamens. Hairy flower bottom.	The serrated edges of the calyx are permanent and remain on the fruit. The fruit is unripe pink or reddish, then dark red to black. Smooth pip
Bark, aroma	The bark smells good. Torn buds smell like almonds.	Torn bark with a characteristic, sharp, relatively pleasant smell, similar to that of blackcurrant.

**Table 3:** Nutrition value and mineral content in *P. padus* i *P. serotina.*

Class	Component	[30]	[31]	[32]	
		*P. padus*	*P. serotina*	*P. serotina*	
		Fruits	Fruits	Seeds Raw	Seeds Toasted
Moisture			81.18 ± 0.081	8.92 ± 0.42	10.75 ± 0.35
Ash			0.86 ± 0.11%	3.19 ± 0.18	2.72 ± 0.21
Mineral	Ca		12.90 ± 1.90	192.30 ± 0.58	127.11 ± 17.51
	Fe			9.49 ± 0.3	1.21 ± 0.003
	Mg		21.20 ± 0.20	249.15 ± 0.34	216.68 ± 18.75
	P		28.10 ± 0.40	439.0 ± 0.16	323.40 ± 0.14
	K		184.30 ± 3.50	873.22 ± 12.64	454.82 ± 0.41
	Zn			3.40 ± 0.10	2.96 ± 0.24
	Na		22.40 ± 1.60	82.98 ± 0.90	23.59 ± 0.8
Protein			2.10 ± 0.01	37 ± 0.16	36.55 ± 0.22
Aminoacid	Asp			112.29 mg/g	116.97 mg/g
	Glu			256.84 mg/g	27.73 mg/g
	Ser			32.84 mg/g	42.11 mg/g
	His			21.60 mg/g	21.29 mg/g
	Gly			37.43 mg/g	38.82 mg/g
	Thr			52.85 mg/g	59.16 mg/g
	Arg			84.24 mg/g	87.42 mg/g
	Ala			41.47 mg/g	44.06 mg/g
	Tyr			48.75 mg/g	60.99 mg/g
	Met			8.93 mg/g	9.83 mg/g
	Val			45.48 mg/g	45.62 mg/g
	Phe			48.64 mg/g	52.00 mg/g
	Ile			39.17 mg/g	40.33 mg/g
	Leu			75.10 mg/g	82.11 mg/g
	Lys			8.85 mg/g	11.17 mg/g
Fat	Total		0.05 ± 0.01	40.37 ± 0.73	39.97 ± 0.20
Carbohydrates		129.28 ± 3.47	12.23 ± 0.79	7.76 ± 2.24	8.65 ± 4.28
Sugars	Glucose	62.19 ± 1.92			
	Fructose	33.34 ± 1.32			
	Sorbitol	33.73 ± 1.51			
Crude Fiber			3.58 ± 0.03	10.73 ± 1.49	12.12 ± 4.06

ND—not detected.

**Table 4:** Phytocompounds content in *P. padus* and *P. serotina.*

Class	Component	Result	Species	Plant Part	Reference
Chlorophyll	Chlorophyll A	42.58 ± 1.92 mg/kg FW	*P. padus*	Fruits	[30]
	Chlorophyll B	22.43 ± 1.30 mg/kg FW	*P. padus*	Fruits	[30]
	Alfa-Caroten	0.05 ± 0.01 mg/kg FW	*P. padus*	Fruits	[30]
	Beta-Caroten	3.06 ± 0.17 mg/kg FW	*P. padus*	Fruits	[30]
Anthocyanins	Cyanidin-2-galactoside	33.69 ± 2.52 mg/kg FW	*P. padus*	Fruits	[30]
	Cyanidin-3-glucoside	1501.53 ± 61.32 mg/kg FW	*P. padus*	Fruits	[30]
		272 mg/100 g	*P. serotina*	Fruits	[41]
	Cyanidin-3-rutinoside	623.68 ± 46.97 mg/kg FW	*P. padus*	Fruits	[30]
	Cyanidin rhamnosyl hexoside	13.09 ± 1.00 mg/kg FW	*P. padus*	Fruits	[30]
Tocopherols	Tocopherol alfa	6.51 ± 0.50 mg/kg FW	*P. padus*	Fruits	[30]
	Tocopherol gama	1.35 ± 0.12 mg/kg FW	*P. padus*	Fruits	[30]
	Tocopherol delta	2.48 ± 0.22 mg/kg FW	*P. padus*	Fruits	[30]
Vitamin C	Ascorbic acid	25.20 ± 3.48 mg/100 FW	*P. padus*	Fruits	[30]
	Dehydroascorbic acid	50.87 ± 16.23 mg/100 FW	*P. padus*	Fruits	[30]
Terpen	Limonene	31.40 ± 5.65 mg/100 FW	*P. padus*	Fruits	[30]
	Phellandrene	8.51 ± 2.69 mg/100 FW	*P. padus*	Fruits	[30]
	Sabinene	1.21 ± 0.18 mg/100 FW	*P. padus*	Fruits	[30]
	γ-terpinene	65.52 ± 6.25 mg/100 FW	*P. padus*	Fruits	[30]
	Ursolic acid	X	*P. serotina*	Fruits	[31]
	Uvaol	X	*P. serotina*	Fruits	[31]
	Cis-Linalool oxide	0.7%	*P. padus*	Flowers	[42]
	Trans s-Linalool oxide	0.1%	*P. padus*	Flowers	[42]
	(Z)-8-Hydroxylinalool	30.4%	*P. padus*	Flowers	[42]
	(E)-β-Farnesene	0.2%	*P. padus*	Flowers	[42]
	(E, E)-α-Farnesene	0.1%	*P. padus*	Flowers	[42]

X—component is present in the sample.

**Table 5:** Polyphenolic compounds in wild cherries (*P. padus* and *P. serotina*).

Class	Component	Result	Species	Plant Part	Reference
Cinnamic acid derivatives	Caffeic acid	6.61 ± 1.35 mg/100g FW	*P. padus*	Fruits	[55]
	Chlorogenic acid	10.48 ± 0.28 mg/100g FW	*P. padus*	Fruits	[55]
		1.39–1.94% DW	*P. padus*	Flowers	[71]
	Coumaric acid	12.20 ± 3.07 mg/100g FW	*P. padus*	Fruits	[55]
	Ferulic acid	10.45 ± 3.65 mg/100g FW	*P. padus*	Fruits	[55]
	5-*p*-Coumaroylquinic acid 1	16.14 ± 1.11 mg/kg FW	*P. padus*	Fruits	[30]
	5-*p*-Coumaroylquinic acid 2	2.25 ± 0.05 mg/kg FW	*P. padus*	Fruits	[30]
	Caffeic acid hexoside1	11.71 ± 1.45 mg/kg FW	*P. padus*	Fruits	[30]
	p-Coumaric acid hexoside 1	10.25 ± 1.00 mg/kg FW	*P. padus*	Fruits	[30]
	5-Caffeoylquinic acid 1	357.30 ± 14.08 mg/kg FW	*P. padus*	Fruits	[30]
	5-Caffeolquinic acid 2	48.25 ± 1.70 mg/kg FW	*P. padus*	Fruits	[30]
		X	*P. serotina*	Fruits	[31]
	Dicaffeoylquinic acid	15.40 ± 1.08 mg/kg FW	*P. padus*	Fruits	[30]
Hydroxy-benzoic Acid derivatives	Ellagic acid	11.41 ± 1.25 mg/100g FW	*P. padus*	Fruits	[55]
	Gallic acid	3.54 ± 0.81 mg/100g FW	*P. padus*	Fruits	[55]
		X	*P. serotina*	Fruits	[31]
	Vanillic acid	X	*P. serotina*	Fruits	[31]
Flavones	Apigenin rhamnoside	24.99 ± 1.61 mg/kg FW	*P. padus*	Fruits	[30]
Flavonols	Hyperoside	7.38 ± 0.41 mg/100g FW	*P. padus*	Fruits	[55]
		0.15–0.23% DW	*P. padus*	Flowers	[71]
	Quercetin	11.86 ± 2.36 mg/100g FW	*P. padus*	Fruits	[55]
		1.37–1.56% DW	*P. padus*	Flowers	[71]
	Quercitrin	16.37 ± 3.51 mg/100g FW	*P. padus*	Fruits	[55]
		X	*P. serotina*	Fruits	[31]
	Rutin	2.67 ± 1.02 mg/100g FW	*P. padus*	Fruits	[55]
		X	*P. serotina*	Fruits	[31]
	Quercetin acetyl hexoside	2.48 ± 0.09 mg/kg FW	*P. padus*	Fruits	[30]
	Quercetin dihexoside	7.49 ± 0.54 mg/kg FW	*P. padus*	Fruits	[30]
	Quercetin hexosyl pentoside 3	223.45 ± 10.20 mg/kg FW	*P. padus*	Fruits	[30]
		X	*P. serotina*	Fruits	[31]
	Quercetin-3-galactoside	52.80 ± 1.41 mg/kg FW	*P. padus*	Fruits	[30]
	Quercetin-3-glucoside	21.40 ± 1.34 mg/kg FW	*P. padus*	Fruits	[30]
	Quercetin-3-rhamnoside	1.98 ± 0.38 mg/kg FW	*P. padus*	Fruits	[30]
	Quercetin-3-rutinosie	64.70 ± 21.18 mg/kg FW	*P. padus*	Fruits	[30]
	Quercetin 3-O-β-galactopyranoside	X	*P. padus*	Flowers	[71]
	Quercetin diglycosides	1.74–1.91% DW	*P. padus*	Spring leaves	[71]
		1.67–1.78% DW	*P. padus*	Flowers	[71]
	Isorhamnetin-3-rutinoside	1.22 ± 0.03 mg/kg FW	*P. padus*	Fruits	[30]
	Isorhamnetin diglycoside	0.36–0.59% DW	*P. padus*	Flowers	[71]
	Kaempferol hexoside pentoside	0.72 ± 0.03 mg/kg FW	*P. padus*	Fruits	[30]
		X	*P. serotina*	Fruits	[31]
	Kaempferol hexoside 1	1.06 ± 0.14 mg/kg FW	*P. padus*	Fruits	[30]
		X	*P. serotina*	Fruits	[31]
	Kaempferol hexoside 2 (glu)	1.43 ± 0.05 mg/kg FW	*P. padus*	Fruits	[30]
		X	*P. serotina*	Fruits	[31]
	Kaempferl-3-rutinoside	2.81 ± 0.29 mg/kg FW	*P. padus*	Fruits	[30]
Catechins	Epicatechin	25.43 ± 3.16 mg/100g FW	*P. padus*	Fruits	[55]
		95.22 ± 10.60 mg/kg FW	*P. padus*	Fruits	[30]
	Catechin	56.66 ± 16.88 mg/100g FW	*P. padus*	Fruits	[55]
Tannins	Castalagin	53.95 ± 8.90 mg/100g FW	*P. padus*	Fruits	[55]
	Vescalagin	26.66 ± 5.97 mg/100g FW	*P. padus*	Fruits	[55]
Organic acids	Citric acid	217.24 ± 14.95 mg/100g FW	*P. padus*	Fruits	[55]
		24.76 ± 1.32 mg/kg FW	*P. padus*	Fruits	[30]
	Oxalic acid	12.16 ± 2.19 mg/100g FW	*P. padus*	Fruits	[55]
	Quinic acid	324.48 ± 57.21 mg/100g FW	*P. padus*	Fruits	[55]
		6.45 ± 0.25 mg/kg FW	*P. padus*	Fruits	[30]
	Malic acid	18.71 ± 0.81 mg/kg FW	*P. padus*	Fruits	[30]
	Shikimic acid	2.66 ± 0.16 mg/kg FW	*P. padus*	Fruits	[30]
	Fumaric acid	80.08 ± 3.19 mg/kg FW	*P. padus*	Fruits	[30]

X—component is present in the sample

**Table 6:** Cyanogenic glycosides: amygdalin content in different species.

Species	Value	Reference
*P. serotina* (leaves)	20.95 ± 0.25 mg/g	[82]
*Prunus amygdalus* Batsch. (fruit)	6.37 mg/g (oil)	[83]
*Prunus amygdalus* L. *(fruit)*	0.12 ± 0.06 mg/g	[85]
*Eriobotrya japonica* Lindl. (flower)	50.76 ± 0.92 µg/mL	[84]
*Prunus armeniaca* L. (raw seeds)	118 (29) µmol HCN equivalents/g DW	[86]
*Amygdalus communis* L. (nectar)	6.7 (ppm)	[87]
*Prunus mume* L. (fruit)	17.49 ± 0.26 mg/g	[85]
*Prunus domestica* L. (fruit)	10.00 ± 0.14 mg/g	[85]
*Prunus persica* L. (peach, fruit)	6.81 ± 0.02 mg/g	[85]
*Prunus avium* L. (red, fruit)	3.89 ± 0.31 mg/g	[85]
*Prunus avium* L. (black, fruit)	2.68 ± 0.02 mg/g	[85]
*Prunus persica* L. (nectarine, fruit)	0.12 ± 0.01 mg/g	[85]

HCN—Hydrogen Cyanide, DW—dry weight.

**Table 7:** In vitro and in vivo activity of *P. padus* and *P. serotina*.

Activity	Method	Extract	Species	Plant Part	Organism	Result/Observed Effect	Reference
Antioxidant Activity	FRAP	Acetone	*P. padus*	Frozen Fruits		31.54 ± 0.26 mM trolox/kg	[30]
	FRAP	Methanol	*P. padus*	Fruits		17.78 ± 0.84 mmol Fe ^2+x^ kg^−1^	[55]
	FRAP	Methanol	*P. padus*	Leaves		100μg/mL (concentration) 0.34 ± 0.04 μg/mL	[88]
	FRAP	Methanol	*P. padus*	Leaves		200 μg/mL (concentration) 0.62 ± 0.01 μg/mL	[88]
	FRAP	Methanol	*P. padus*	Leaves		300 μg/mL (concentration) 0.88 ± 0.00 μg/mL	[88]
	FRAP	Methanol	*P. padus*	Branch		100μg/mL (concentration) 0.51 ± 0.01	[88]
		Methanol	*P. padus*	Branch		200 μg/mL (concentration) 0.74 ± 0.01	[88]
		Methanol	*P. padus*	Branch		300 μg/mL (concentration) 0.99 ± 0.00	[88]
	FRAP	Water	*P. serotina*	Fresh Fruits		1455.2 ± 92.5 μmol TE/100 g of FW	[31]
	FRAP	Water	*P. serotina*	Flesh Fruits		1100.7 ± 35.4 μmol TE/100 g of FW	[31]
	FRAP	Water	*P. serotina*	Peel Fruits		1991.4 ± 40.1 μmol TE/100 g of FW	[31]
	DPPH	Methanol	*P. padus*	Flowers		1.43–1.49 g g^−1^	[71]
	DPPH	Methanol	*P. padus*	Autumn Leaves		1.68 g ^g−1^	[71]
	DPPH	Methanol	*P. padus*	Spring Leaves		2.10–2.29 g g^−1^	[71]
	DPPH	Methanol	*P. padus*	Summer Leaves		1.81–1.93 g g^−1^	[71]
	DPPH	Methanol	*P. padus*	Flowers		1.78–1.84 g g^−1^	[71]
	DPPH	Methanol	*P. padus*	Autumn Leaves		2.49 g g^−1^	[71]
	DPPH	Methanol	*P. padus*	Spring Leaves		4.27–4.92 g g^−1^	[71]
	DPPH	Methanol	*P. padus*	Summer Leaves		3.12–3.95 g g^−1^	[71]
	DPPH	Water	*P. serotina*	Fresh Fruits		2056.7 ± 108.0 μmol TE/100 g of FW	[31]
	DPPH	Water	*P. serotina*	Flesh Fruits		1764.6 ± 170.4 μmol TE/100 g of FW	[31]
	DPPH	Water	*P. serotina*	Peel Fruits		2681.6 ± 180.0 μmol TE/100 g of FW	[31]
Antimicrobial	MIC	Methanol	*P. padus*	Branch	*Bacillus Atrophaeus*	250 μg/mL	[89]
	MIC	Methanol	*P. padus*	Leaves	*Bacillus Atrophaeus*	500 μg/mL	[89]
	MIC	Methanol	*P. padus*	Branch	*Kocuria rhizophila*	125 μg/mL	[89]
	MIC	Methanol	*P. padus*	Leaves	*Kocuria rhizophila*	>1000 μg/mL	[89]
	MIC	Methanol	*P. padus*	Branch	*Micrococcus luteus*	>1000μg/mL	[89]
	MIC	Methanol	*P. padus*	Leaves	*Micrococcus luteus*	>1000 μg/mL	[89]
	MIC	Methanol	*P. padus*	Branch	*Staphylococcus epidermidis*	250 μg/mL	[89]
	MIC	Methanol	*P. padus*	Leaves	*Staphylococcus epidermidis*	>1000 μg/mL	[89]
	MIC	Methanol	*P. padus*	Branch	*Bacillus subtilis*	500 μg/mL	[89]
	MIC	Methanol	*P. padus*	Leaves	*Bacillus subtilis*	500 μg/mL	[89]
	MIC	Methanol	*P. padus*	Branch	*Klebsiella pneumoniae*	1000 μg/mL	[89]
	MIC	Methanol	*P. padus*	Leaves	*Klebsiella pneumoniae*	>1000 μg/mL	[89]
	MIC	Methanol	*P. padus*	Branch	*Enterobacter cloacae*	500 μg/mL	[89]
	MIC	Methanol	*P. padus*	Leaves	*Enterobacter cloacae*	>1000 μg/mL	[89]
	MIC	Methanol	*P. padus*	Branch	*Salmonella enterica*	500 μg/mL	[89]
	MIC	Methanol	*P. padus*	Leaves	*Salmonella enterica*	>1000 μg/mL	[89]
	MIC	Methanol	*P. padus*	Branch	*Pseudomonas aeruginosa*	1000 μg/mL	[89]
	MIC	Methanol	*P. padus*	Leaves	*Pseudomonas aeruginosa*	1000 μg/mL	[89]
	MIC	Dichloromethane	*P. padus*	Seeds	*Enterococcus faecalis*	1.0 mg/mL	[89]
	MIC	Methanol	*P. padus*	Seeds	*L. plantarum*	1.0 × 10^−2^ mg/mL	[89]
	MIC	Methanol	*P. padus*	Seeds	*P. mirabilis*	1.0 × 10^−2^ mg/mL	[89]
	MIC	Methanol	*P. padus*	Seeds	*S. aureus*	1.0 × 10^−4^ mg/mL	[89]
	MIC	Dichloromethane	*P. padus*	Seeds	*S. hominis*	1.0 mg/mL	[89]
	MIC	Methanol	*P. padus*	Seeds	*S. hominis*	1.0 × 10^−2^ mg/mL	[89]
Antidiabetic	The α-glucosidase inhibitory effect	Methanol	*P. padus*	Leaves		1.0 ± 0.1 µg/mL	[88]
	The α-glucosidase inhibitory effect	Methanol	*P. padus*	Branch		82.7 ± 4.2 µg/mL	[88]
Cardiovascular activity	NOS Enzymatic activity assay	Dichloromethane	*P. serotina*	Fruits	Wistar male rats 250–300 g	Ursolic acid (EC_50_ = 21.5 ± 3.5 µg/mL; Emax = 97.7% ± 3.9%); Uvaol (EC_50_ = 19.3 ± 2.5 µg/mL; Emax = 93.4% ± 5.1%) caused a significant concentration-dependent relaxation of the aorta. ACh (EC_50_ = 8.7 ± 0.8 µg/mL; Emax = 69.5% ± 5.7%), used as positive control, was more potent than ursolic acid and uvaol, but showed lower efficacy than both triterpenes. The nitrite concentration increased, when aortic tissue was incubated with ursolic acid ((NO2′) = 7.95 ± 0.22µM) and uvaol ((NO2′) = 7.55 ± 0.17µM); both triterpenes induced a higher nitrite concentration than that of ACh ((NO2’) = 5.5 ± 0.47µM). Similarly, quantification of H_2_S showed that stimulation of aortic tissue with ursolic acid ((H_2_S) = 234 ± 12.7µM) and uvaol ((H_2_S) = 253 ± 6.8µM) increased four times the H2S concentration with respect to the control. In the presence of ACh, H_2_S levels were only three times higher than those of the control; the vasodilator effect produced by ursolic acid involves activation of the NO/cGMP and H2S/KATP channel pathways, possibly through direct activation of NOS and CSE.	[90]
Anti-inflammatory-nociceptive properties	Measurement of iNOS enzyme activity Western blot analysis Trypsin-induced paw edema Acute toxicity test Tail immersion test Hot plate test Acetic acid-induced writhing test Formalin test	Methanol	*P. padus*	Dried stem	ICR mice (6 weeks old) weighing 20–25 g and C57BL/6 mice (5 weeks old) weighing 18–22 g	Tested material delayed reaction times to a nociceptive stimulus 60 min after oral administration (38.61% at 250 mg/kg and 68.51% at 500 mg/kg, po0.001).The anti-nociceptive effects of MPP (250, 500 mg/kg) occurred between 30 and 90 min and maximum analgesia was reached at 60 min (37.98%, after 0.01 and 62.18%, after 0.001 respectively).The treatment with MPP induced a significant decrease in the number of writhing motions dose dependently (52.5% at 250 mg/kg, po0.001 and 72.8% at 500 mg/kg, po0.001). *P. padus* shown anti-inflammatory properties not only by suppressing various inflammatory mediators in vitro but also by reducing inflammatory swelling in vivo. A strong anti-nociceptive effect through the central and peripheral mechanism acting as a partial opioid agonist was also demonstrated.	[91]

DPPH—antioxidant activity with DPPH radicals (2,2-diphenyl-1-picrylhydrazyl), FRAP—ferric reducing antioxidant power, MIC—antimicrobial test (minimum inhibitory concentration assay). TE—Trolox equivalent, FW—fresh weight, NOS—inhibitor of NO synthase, CSE—inhibitor of cystathionine-γ-lyase, ICR—Institute of Cancer Research.
